# Distance-Entropy: An Effective Indicator for Selecting Informative Data

**DOI:** 10.3389/fpls.2021.818895

**Published:** 2022-01-13

**Authors:** Yang Li, Xuewei Chao

**Affiliations:** ^1^College of Mechanical and Electrical Engineering, Shihezi University, Xinjiang, China; ^2^School of Electrical and Information Engineering, Tianjin University, Tianjin, China

**Keywords:** quality assessment, agriculture, pest, entropy, few-shot

## Abstract

Smart agriculture is inseparable from data gathering, analysis, and utilization. A high-quality data improves the efficiency of intelligent algorithms and helps reduce the costs of data collection and transmission. However, the current image quality assessment research focuses on visual quality, while ignoring the crucial information aspect. In this work, taking the crop pest recognition task as an example, we proposed an effective indicator of distance-entropy to distinguish the good and bad data from the perspective of information. Many comparative experiments, considering the mapping feature dimensions and base data sizes, were conducted to testify the validity and robustness of this indicator. Both the numerical and the visual results demonstrate the effectiveness and stability of the proposed distance-entropy method. In general, this study is a relatively cutting-edge work in smart agriculture, which calls for attention to the quality assessment of the data information and provides some inspiration for the subsequent research on data mining, as well as for the dataset optimization for practical applications.

## Introduction

Smart agriculture is established based on the digital process, combining the data in the agricultural field and the Information and Communications Technology (ICT) (Friha et al., 2021; Sun et al., 2021). As for the intelligent plant protection, the related data have various sources, such as the sensing of soil (Yin et al., 2021), light intensity (Yu et al., 2021), water stress (Mundim and Pringle, 2018; Ihuoma and Madramootoo, 2019), mixture of water and fertilizer (Jia et al., 2019), temperature and humidity (Mekala and Viswanathan, 2019), etc. However, more commonly used data sources in artificial intelligence (AI)-driven applications are images or videos. For example, based on the RGB image processing or hyperspectral image processing, there have been numerous typical studies and applications, including agricultural yield forecasting (Khaki and Wang, 2019; Shahhosseini et al., 2020; Jarlan et al., 2021), crop pests and diseases identification (Li and Chao, 2020; Li et al., 2020; Liu and Wang, 2020; Liang, 2021), agricultural robot and navigation (Wen et al., 2020; Zhang et al., 2020; Emmi et al., 2021), counting of plant fruits (Lin and Guo, 2020; Fu et al., 2021), etc.

Deep learning is the primary implementation of intelligent applications by combining ICT and agriculture, but the shortcomings are also apparent. One main drawback is that deep models are unfriendly to a small amount of data and have a severe over-fitting problem. However, the limited amount of data is the essential property of many real-world tasks. Even in scenarios with big data, there is also an inevitable problem in the early stages of data collection.

Considering the execution of AI-based algorithms, they are mainly cloud computing and edge computing. For cloud computing (Zuo et al., 2016; Zhu et al., 2017), the required communication bandwidth of data transmission is very high for real-time performance. The data quality should be treated seriously as this is gradually becoming a research focus, thus, helping to improve the training efficiency of models. On the other hand, edge computing (Yang et al., 2018; Huang et al., 2021) has relatively weak hardware resources and high training costs. Therefore, the data quality analysis and screening are significant for practical applications.

To explore the effect of data quality, a machine learning based on limited data, also called few-shot learning, has made some attempts and has emerged in many scenarios (Li and Yang, 2020, 2021; Chao and Zhang, 2021; Li and Chao, 2021; Li et al., 2021; Nie et al., 2021). But, most of the existing related studies in the literature are based on the randomly selected few data, without enough consideration of data information value. The related research works are mainly meta-learning, model fine-tuning, and applications (Karami et al., 2020; Nuthalapati and Tunga, 2021; Yang Y. et al., 2021; Zhou et al., 2021). Therefore, the small amount of data must be built on the premise of high quality to be meaningful. However, the current image quality assessment (IQA) research works are mainly focused on visual evaluation, including screen images (Yang et al., 2020; Yang J. et al., 2021) and stereoscopic video (Yang et al., 2019; Zhao et al., 2021). That is to say, there are limited research on image quality and information assessment aiming at AI-driven visual tasks.

To fill this gap, in this paper, we proposed an innovative and effective indicator called distance-entropy, to assess data quality for the machine vision recognition tasks in agriculture. In particular, the proposed distance-entropy indicator is used to select informative samples or redundant data. The crop pest dataset is used and analyzed under different mapping feature dimensions and base data sizes to verify the validity of the proposed indicator. Extensive experiments showed that the distance-entropy indicator can be used to distinguish good and bad data from the perspective of information. Thus, this study can assess and screen high-quality data in an existing dataset and guide the high informative online data gathering to establish a high-quality dataset.

The rest of this article is arranged as follows. Section Materials and Methods describes the used dataset, quality assessment based on feature distribution, and the proposed distance-entropy method. In Section Results, many experiments and result visualization are carried out, considering the factors of mapping feature dimensions and the base data size. Finally, Discussion and Conclusion are put in Section 4 and Section 5, respectively.

## Materials and Methods

### Dataset

This study adopted the plant pest recognition as the target task, an essential part of intelligent plant protection. The used plant pest dataset has six classes. There are 1,000 image samples in each category, with a uniform size of 224^*^224^*^3. Differently from other datasets under the controlled environmental background, the colorful RGB images in this dataset are collected in the natural environment. The dataset will be split into training and testing sets, and the training set will be split into base and pool data.

**Figure 1:**
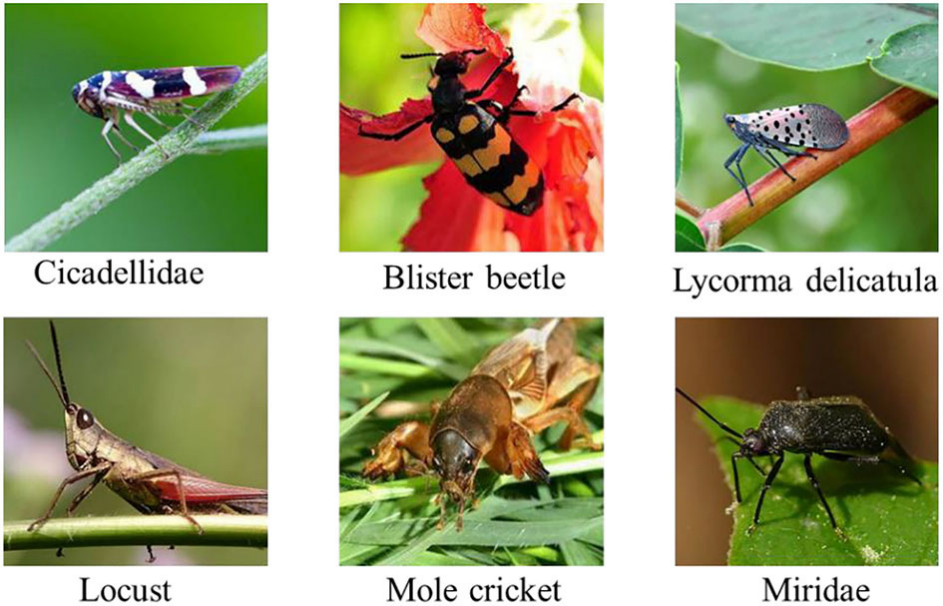
Some image samples of the pest dataset.

The details of the category name and data quantity are shown in Table 1, while some image samples are shown in Figure 1. Notably, the symbol N is a variable used to verify the effectiveness and robustness of the proposed distance-entropy indicator.

**Table 1:** Details of the used pest dataset.

**Pest dataset**	**Name**	**Base data size**	**Pool data size**	**Test data size**
Category 1	Cicadellidae	N	800-N	200
Category 2	Blister beetle	N	800-N	200
Category 3	Lycorma delicatula	N	800-N	200
Category 4	Locust	N	800-N	200
Category 5	Mole cricket	N	800-N	200
Category 6	Miridae	N	800-N	200

### Image Quality Assessment

**Figure 2:**
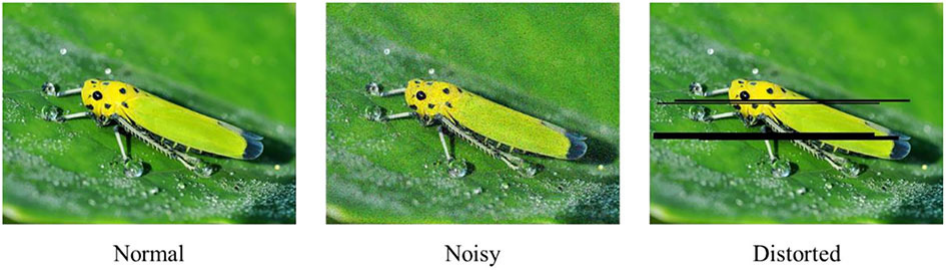
Images with different visual qualities.

The existing IQA focuses on the image quality evaluation at the aspects of transmission distortion and pixels imaging. For example, one typical pest image, its noisy image, and the corresponding transmission distortion image are shown in Figure 2. Furthermore, the current IQA studies aim to design some algorithms to automatically provide a score in assessing the visual image quality, and the main goal is to approximate the subjective score given by human volunteers.

**Figure 3:**
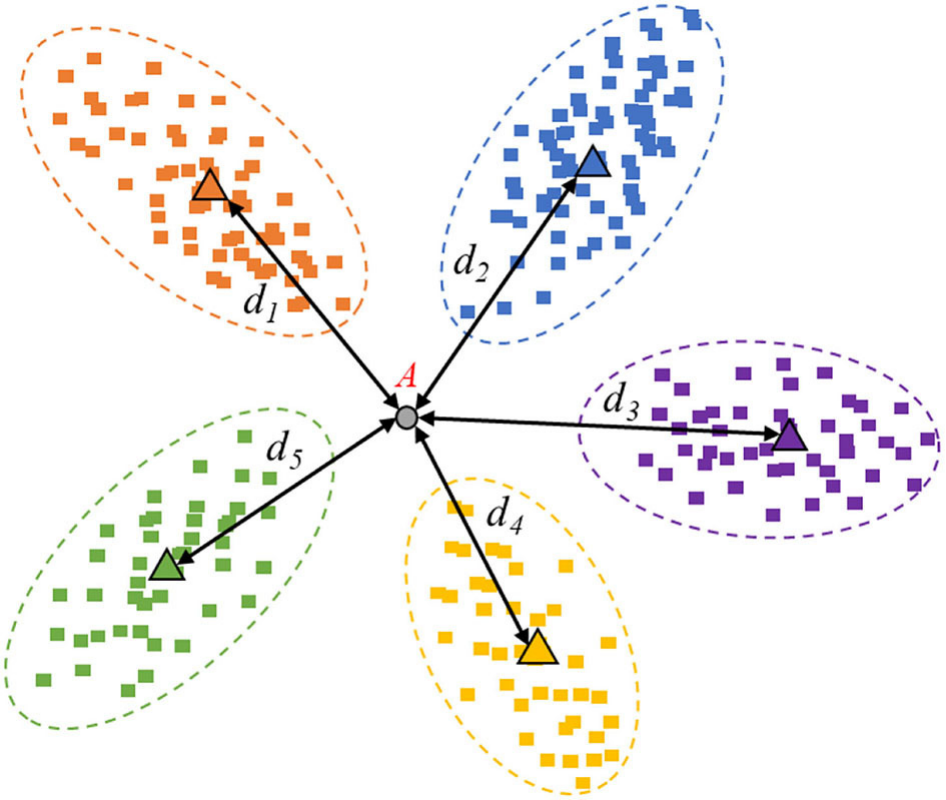
Image example with high information quality.

**Figure 4:**
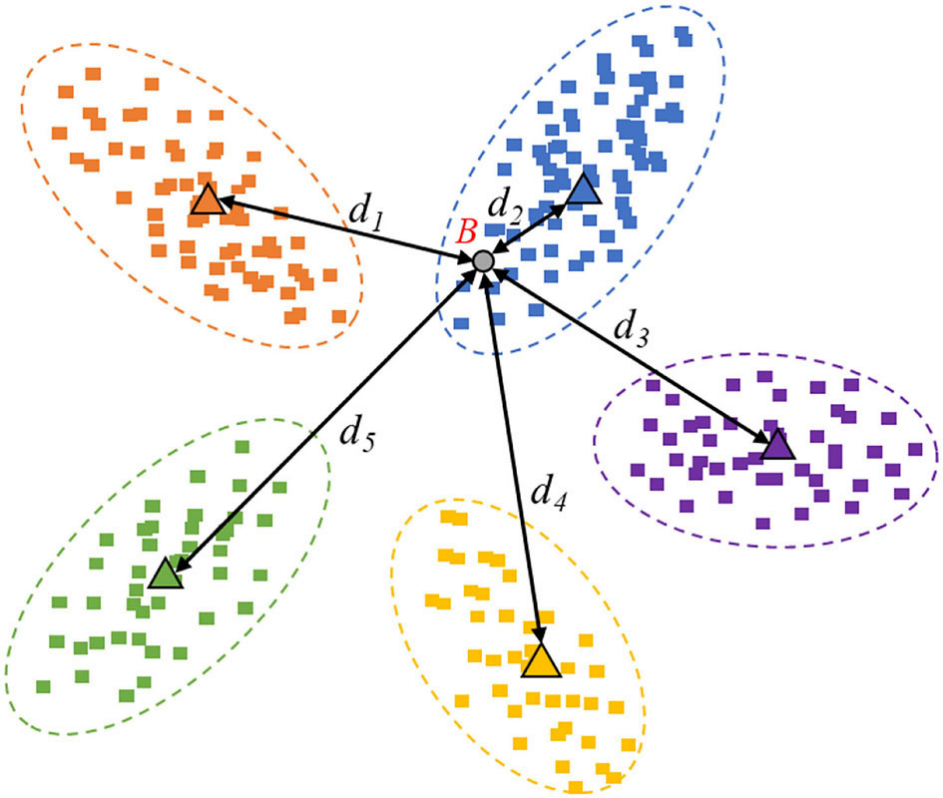
Image example with low information quality.

However, in this study, we are not concerned about the visual image quality, but rather about the image information quality. In particular, taking the multi-classification identification task as an example, the mapping feature space can be visualized as Figures 3, 4.

In Figure 3, sample “A” is an example of valuable data with high information quality, which is helpful to the performance improvement. The high-informative data can provide information that the currently collected data does not include. Hence, the spatial mapping feature belongs to the common area and cannot fall into the existing range of categories due to the uncertain prediction.

In Figure 4, sample “B” is an example of redundant data with low information quality, which is meaningless to the performance improvement. The low informative data cannot provide new information that the collected data does not include. Hence, the spatial mapping position falls into the existing range of categories due to the confident prediction.

### Distance-Entropy Indicator

In this work, we proposed an effective indicator called distance-entropy, where the distance is used to measure similarity, and the entropy is used to evaluate the information. Specifically, take Figure 3 as an example. The feature distributions stand for the existing base data used to train the current model. First, the prototypes of categories are computed. Then, the Euclidean distance between the new sample and the class prototype is calculated as *di*. Next, the Euclidean distances will be changed to a proportional distribution based on the softmax function, written as Equation 1. Finally, the information entropy *E* is calculated based on Equation 2, according to the proportional distribution coming from the distances.


(1) $$\begin{array}{r} S\left(d_{i}\right)=\frac{e^{d_{i}}}{\underset{i}{\sum }e^{d_{j}}} \end{array}$$

(2) $$\begin{array}{r} E=-\underset{i}{\sum }S\left(d_{i}\right)\cdot {\text{log}}_{2}S\left(d_{i}\right) \end{array}$$

In practice, the distances between new samples and prototypes can be multiplied by−1. The smaller distance represents a more considerable similarity and should have greater weight in the proportional transformation based on the softmax function, which is also more interpretable. As known, the Maximum Entropy Theorem indicates that when all the probability values are equal, the information entropy of this event will be maximum. Since the event is the most chaotic, no clear bias can be given. Comparing Figure 3 with Figure 4, the sample in Figure 3 is high informative, whose distance-entropy will be much larger than the one in Figure 4, which has a definite classification bias.

## Results

To verify the effectiveness and robustness of the proposed distance-entropy indicator, we conducted many groups of experiments, considering the factors of mapping feature dimensions and base data size. The experiments were implemented based on a graphic processing unit (GPU) of NVIDIA TITAN Xp, with a 12 GB memory. The distance-entropy algorithm was running on the Jupyter Notebook using Python, with libraries of TensorFlow, Keras, and Numpy.

### Effect of Mapping Feature Dimensions

The different mapping feature dimensions represent different feature spaces, and we compared three mapping dimensions of 2, 64, and 128. The base data were 50 samples per class. The selected data, according to the high distance-entropy and low distance-entropy, were added to the original base data and used to refine the model. Finally, the fixed test data were used to compare the performance of accuracy. Notably, the number of selected data in each category is fixed as 10 for comparative analysis in each step.

The results of accuracy testing under different mapping feature dimensions are shown in Table 2, and the symbol “d-e” is short for distance-entropy.

**Table 2:** Results under different mapping feature dimensions.

**Accuracy (%)**	**2 dimensions**		**64 dimensions**		**128 dimensions**	
	**High d-e**	**Low d-e**	**High d-e**	**Low d-e**	**High d-e**	**Low d-e**
Base data	82.6	82.6	90.3	90.3	90.3	90.3
Add 10	87.5	83.6	93.1	90.6	92.6	90.8
Add 20	87.7	84.8	93.5	91.1	93.1	91.4
Add 30	87.9	85.7	94.6	91.8	94.4	92.2
Add 40	89	86.7	95.2	92.1	95	92.4
Add 50	89.7	87	95.6	92.2	95.5	92.3

**Figure 5:**
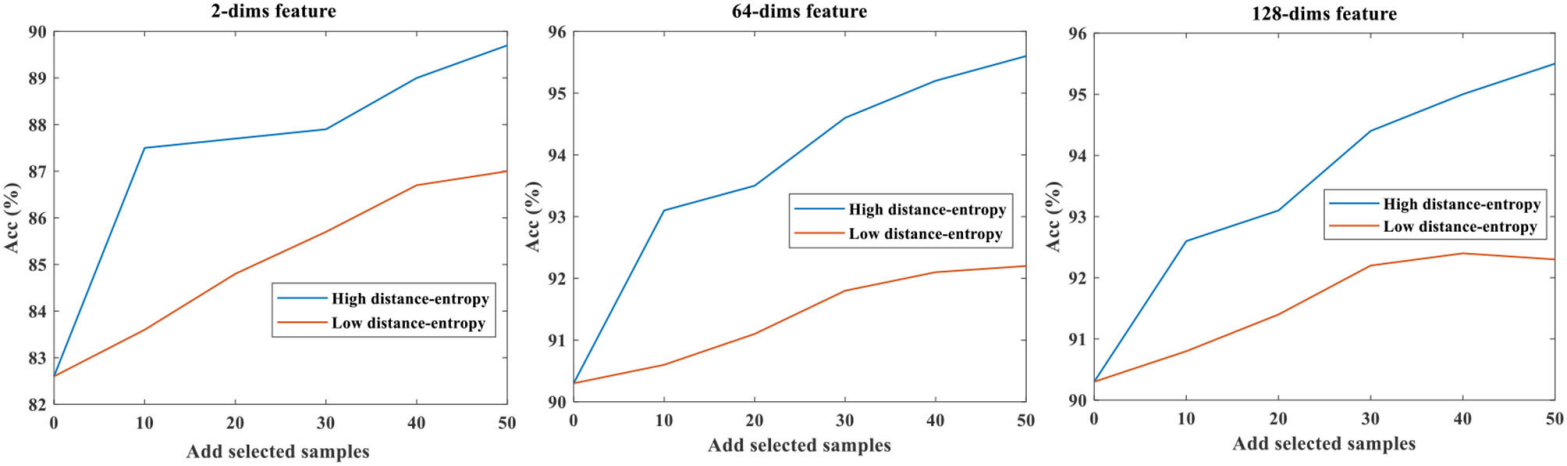
The accuracy testing under different mapping feature dimensions.

Evidently, the proposed distance-entropy method is effective under different mapping dimensions and is able to distinguish the high informative data from the low informative data, which are reflected in the improvement of task performance. Notably, the “Add 30” is compared to the “Base data,” which means adding 10 samples to the “Add 20.” The results can also be shown in Figure 5, which are easier to present the consistency of differences in selecting data samples.

As shown, the comparative trends in Figure 5 are consistent, while the upper accuracies are different because the mapping dimensions are related to the representative ability of the model. Thus, considering the effect of mapping dimensions, the distance-entropy can be a reliable and efficient indicator for selecting high-information samples.

### Effect of Base Data Size

The base data represented the existing data used to train the model for recognition or for other tasks. The different base data sizes will affect the performance of the model and the corresponding feature distributions. To verify the validity of the proposed distance-entropy indicator, we conducted comparative experiments with the base data of 50 and 100 samples per class. Since the impact analysis of mapping dimensions has been carried out in section 3.1, only two dimensions are taken as an example. Different samples were selected, added to the original base data, and then used to refine the model according to high and low distance-entropy indicators. As before, the number of selected data per category is fixed as 10 for comparative analysis. The results of testing accuracy under different base data sizes are shown in Table 3, and the symbol “d-e” is short for distance-entropy.

**Table 3:** Results under different base data sizes.

	**Base data**		**Base data**	
**Accuracy (%)**	**50 samples per class**		**100 samples per class**	
	**High d-e**	**Low d-e**	**High d-e**	**Low d-e**
Base data	82.6	82.6	87.7	87.7
Add 10	87.5	83.6	90.5	88.4
Add 20	87.7	84.8	92.1	91.2
Add 30	87.9	85.7	92.8	91.5
Add 40	89	86.7	93	91.8
Add 50	89.7	87	93.3	92

Similar to the trends in section Effect of Mapping Feature Dimensions, the proposed distance-entropy indicator is effective under the different base data sizes to distinguish high and low informative samples, as reflected in the performance improvement of the model.

**Figure 6:**
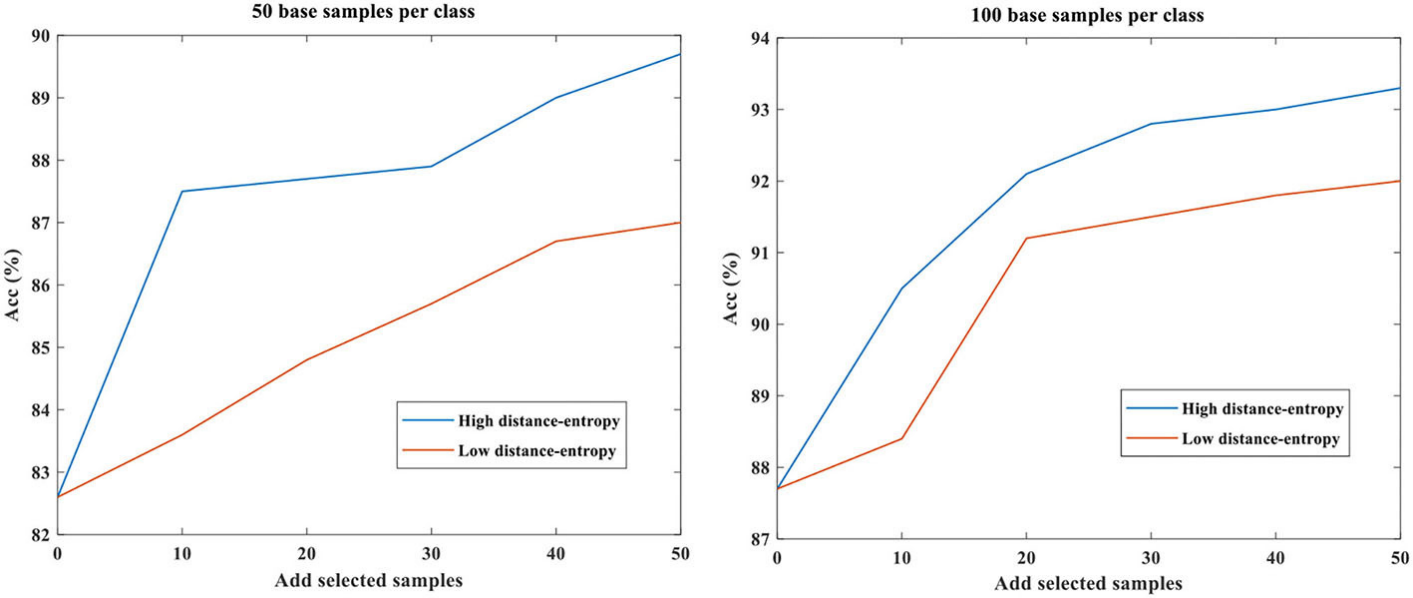
The testing accuracy under different base data sizes.

The results are plotted in Figure 6, showing the consistency of trends.

As seen, the accuracies at “Add 50” are different under different conditions; the larger the base data size, the better the performance of the model is. Then, the addition of high informative data will bring a big promotion, whereas the addition of low informative data will only bring little improvement. Thus, the accuracy in case of large base data with high distance-entropy is the largest, i.e., 93.3%. Even so, the comparison trends of selection results according to the distance-entropy indicator are consistent, showing the ability to distinguish high and low informative data.

### Visualization of Different Selected Results

**Figure 7:**
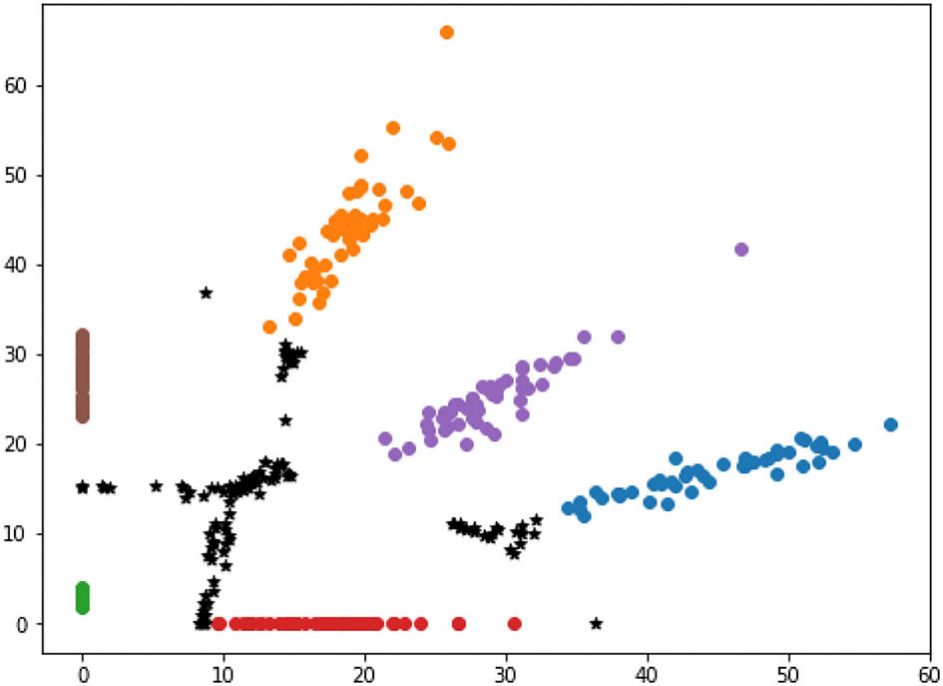
The visualization of high distance-entropy samples.

**Figure 8:**
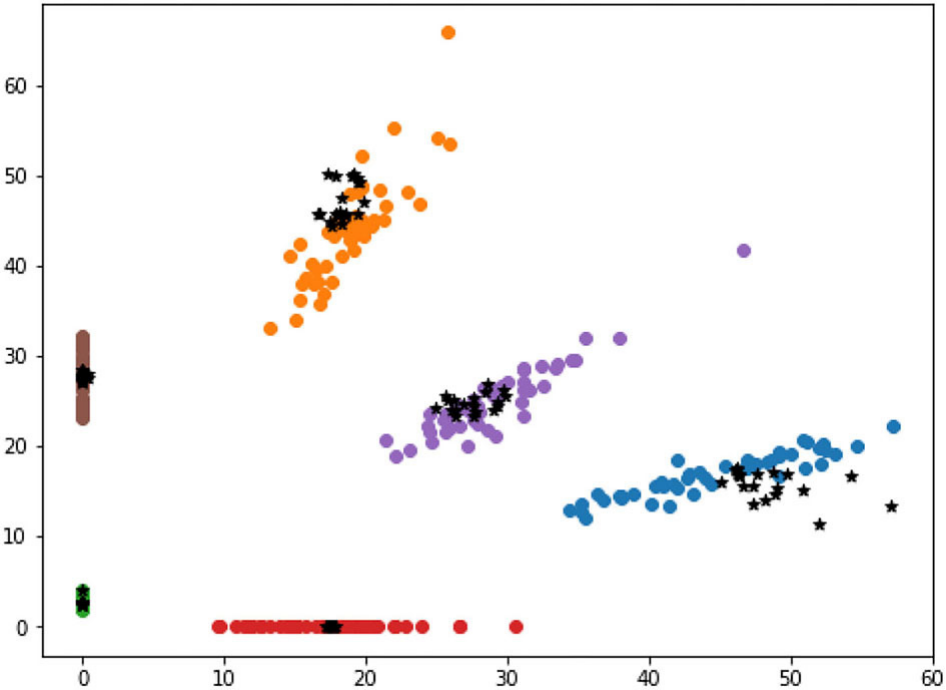
The visualization of low distance-entropy samples.

This section compares the selected samples according to high and low distance-entropy, using two dimensions visualization to intuitively display the distribution of the selected results with different information quality. In addition, due to the dimension requirements of visualization, the features can be directly mapped to two dimensions or processed by t-Distributed Stochastic Neighbor Embedding (t-SNE) dimension reduction. The visualization of selected samples according to high and low distance-entropy are shown in Figures 7, 8, respectively.

In Figure 7, the black points stand for the selected samples according to high distance-entropy, while other colorful points represent the existing base data from six categories. It is shown that the selected data are located at the central area among all the categories, and that there are no overlaps. The visualization results show that the selected samples with high distance-entropy can provide more information that is unavailable to the existing base data. Thus, these samples are good data from the perspective of the information quality assessment.

In Figure 8, the black points are the selected samples according to low distance-entropy, which are almost overlapped with the existing base data. Indeed, this kind of sample cannot provide helpful information for the pattern recognition tasks. Thus, the selected samples according to low distance-entropy can be regarded as bad data from the perspective of information quality assessment.

## Discussion

This work focused on the data information quality assessment, carried out many comparative experiments, and analyzed the visualization results. In this section, the motivations, contributions, reasons, limitations, and future work of this study are discussed.

### Motivations

In intelligent plant protection, smart agriculture, and other real-world applications, the long-tailed data distribution is a basic fact, which means the cost of obtaining rare data is high. As for the current data-driven intelligent algorithms, data quality assessment is critical and necessary because a large number of redundant and low-quality data can waste the data transmission and does not help improve the task performance.

Despite such a crucial need, the current IQA research are primarily at the visual level, such as compression, transmission, and consistency with subjective visual evaluation. However, from our viewpoint, these visual IQA works are meaningful but not sufficiently in-depth. Particularly, it is important to conduct information quality evaluation and consider the requirements of a data-driven intelligent algorithm. Thus, we proposed the distance-entropy indicator to distinguish the data quality.

### Contributions

The core contribution of this paper is the proposed distance-entropy indicator. Taking the spatial distribution of the mapping feature into account, it can be used to evaluate the information value of new samples. Here, the new samples refer to online data collecting. In addition, the existing dataset can also be split into two parts: one is the base data, whereas the other can be regarded as new samples to analyze the data quality and optimize the dataset.

Extensive experiments prove the stability and reliability of the proposed distance-entropy indicator, which is neither affected by the feature dimension nor the base data size. The indicator can be used to distinguish high and low informative samples. Furthermore, the proposed distance-entropy method can provide some inspiration for new data gathering and dataset optimization in the field of intelligent plant protection, such as remote sensing data collection, plant status monitoring, and plant disease identification.

### Reasons

The spatial distribution of mapping feature corresponds to the unique attributes of data. If a new sample is informative and different, the intelligent model has weak confidence and will be mapped to the common area among all the classes. An intuitive explanation is that the model also does not know which category it belongs to (refer to the cases in Figure 7).

Furthermore, supposing one new sample is redundant; In that case, its mapping feature must be close to some original sample because the intelligent model has already been familiar with it, mapped to the area which is overlapped with the existing base data (refer to the cases in Figure 8).

Finally, the proposed distance-entropy indicator can implement the information quality assessment because the distance-entropy value is calculated based on the relations among the new sample and all the categories. When the distance-entropy is large, the degree of chaos is high, i.e., the model does not know which category the sample belongs to. But, when the distance-entropy is small, the degree of chaos is low, i.e., the model confidently knows which category the sample belongs to.

### Limitations and Further Work

This work adopted the Euclidean distance, measuring the similarity between a new sample and various distributions of base data, which is an easy and fast way to perform. However, this measure only considers the prototype calculated by average and cannot take care of some scattered data cases. We would further analyze other suitable metric methods in the further work and combine them with the proposed distance-entropy indicator. This study used multi-classes pest recognition as an example. Hence, in succeeding work, we would consider other vision tasks and testify the validity of the proposed distance-entropy indicator.

## Conclusion

To evaluate data quality at the informative level, we proposed a novel indicator of distance-entropy to distinguish the high and low informative data. Taking crop pest identification as an example, many comparative experiments, considering factors of different mapping feature dimensions and base data sizes, were conducted to testify the validity and robustness. The results show that the proposed distance-entropy method can reliably and efficiently distinguish good and bad data from the informative perspective. The comparison trends remain consistent under different experimental conditions, showing adaptability. In general, this study is a relatively cutting-edge research work in the field of intelligent agriculture. It can provide some inspiration for the data information assessment and lay a foundation for the subsequent data assessment and dataset optimization.

## Data Availability Statement

The data analyzed in this study is subject to the following licenses/restrictions: Requests to access these datasets should be directed to Xuewei Chao, sherry_chao@shzu.edu.cn.

## Author Contributions

YL contributed to the conception and design of the study and wrote the first draft of the manuscript. XC organized the database and performed the statistical analysis. All authors contributed to manuscript revision, read, and approved the submitted version.

## Funding

This work was supported by the National Natural Science Foundation of China (No. 32101612) and the Major Science and Technology Projects of Xinjiang Production and Construction Corps (No. 2021AA006).

## Conflict of Interest

The authors declare that the research was conducted in the absence of any commercial or financial relationships that could be construed as a potential conflict of interest.

## Publisher's Note

All claims expressed in this article are solely those of the authors and do not necessarily represent those of their affiliated organizations, or those of the publisher, the editors and the reviewers. Any product that may be evaluated in this article, or claim that may be made by its manufacturer, is not guaranteed or endorsed by the publisher.
